# Association of object mnemonic discrimination deficit with age and AD biomarkers in older adults

**DOI:** 10.64898/2026.09.14.750966

**Published:** 2026-09-17

**Authors:** Zhengshi Yang, Xiaowei Zhuang, Lynn M. Bekris, Maria Khrestian, Katherine A. Koenig, Tim Curran, Rajesh Nandy, Pavithran Pattiam Giriprakash, Chendi Han, Jagan Pillai, Robert Fox, Mark J. Lowe, Dietmar Cordes

## Abstract

**Background:**

Blood–based Alzheimer’s disease (AD) biomarkers provide scalable detection of AD pathology, yet their relationships with hippocampal–dependent mnemonic discrimination remain unclear. This study examined how object mnemonic discrimination relates to age, hippocampal structure, and plasma AD biomarkers in cognitively normal (CN) older adults and individuals with amnestic mild cognitive impairment (aMCI).

**Methods:**

Forty–six CN and forty–one aMCI participants completed an object mnemonic similarity task (MST). Mnemonic discrimination was quantified using the overall lure discrimination index (LDI) and similarity–specific LDIs. Plasma pTau181, pTau217, pTau231, and GFAP were assayed, and hippocampal volumes were derived from 7T MR scanner. Multiple linear regression models assessed associations between LDI and biomarkers, controlling for age, sex, education, APOE4 status, and diagnosis. Repeated–measures ANCOVA and linear mixed effects models evaluated similarity–dependent age effects. ROC analyses and stepwise logistic regression quantified diagnostic classification performance.

**Results:**

LDI was significantly reduced in aMCI compared to CN. Total hippocampal volume and plasma pTau217 showed the strongest associations with LDI, exceeding those of pTau181, pTau231, and GFAP. LDI discriminated aMCI from CN with high accuracy (AUC = 0.836), comparable to hippocampal volume and plasma biomarkers. A combined model including LDI, pTau181, pTau217, and hippocampal volume achieved the highest diagnostic accuracy (AUC = 0.931). Age effects were similarity–dependent: discrimination declined with age only for low–similarity lures, independent of diagnosis. Sex differences emerged within aMCI, with females showing lower LDI than males.

**Conclusions:**

Object mnemonic discrimination provides a sensitive behavioral marker linked to hippocampal volume and plasma pTau217, showing discriminative power comparable to established AD biomarkers in separating aMCI from CN participants. Similarity–dependent age effects highlight age-related deficits are not universal for lures with varying similarity levels.

## Full Text Availability

The license terms selected by the author(s) for this preprint version do not permit archiving in PMC. The full text is available from the preprint server.

